# Cystic Fibrosis Rapid Response: Translating Multi-omics Data into Clinically Relevant Information

**DOI:** 10.1128/mBio.00431-19

**Published:** 2019-04-16

**Authors:** Ana Georgina Cobián Güemes, Yan Wei Lim, Robert A. Quinn, Douglas J. Conrad, Sean Benler, Heather Maughan, Rob Edwards, Thomas Brettin, Vito Adrian Cantú, Daniel Cuevas, Rohaum Hamidi, Pieter Dorrestein, Forest Rohwer

**Affiliations:** a Department of Biology, San Diego State University, San Diego, California, USA; b Viral Information Institute at San Diego State University, San Diego, California, USA; c Skaggs School of Pharmacy, University of California San Diego, La Jolla, California, USA; d Department of Medicine, Division of Pulmonary, Critical Care and Sleep Medicine, University of California San Diego, La Jolla, California, USA; e Ronin Institute, Montclair, New Jersey, USA; f Wholon, San Diego, California, USA; g Computational Sciences Research Center, San Diego State University, San Diego, California, USA; h Argonne National Laboratory, Argonne, Illinois, USA; UT Southwestern Med Center Dallas; University of Minnesota; Arizona State University

**Keywords:** Shiga toxins, clinical metagenomics, cystic fibrosis, metabolomics, metatranscriptome

## Abstract

Pulmonary exacerbations are the leading cause of death in cystic fibrosis (CF) patients. To track microbial dynamics during acute exacerbations, a CF rapid response (CFRR) strategy was developed. The CFRR relies on viromics, metagenomics, metatranscriptomics, and metabolomics data to rapidly monitor active members of the viral and microbial community during acute CF exacerbations. To highlight CFRR, a case study of a CF patient is presented, in which an abrupt decline in lung function characterized a fatal exacerbation. The microbial community in the patient’s lungs was closely monitored through the multi-omics strategy, which led to the identification of pathogenic shigatoxigenic Escherichia coli (STEC) expressing Shiga toxin. This case study illustrates the potential for the CFRR to deconstruct complicated disease dynamics and provide clinicians with alternative treatments to improve the outcomes of pulmonary exacerbations and expand the life spans of individuals with CF.

## Abstract (precis)

Proper management of polymicrobial infections in patients with cystic fibrosis (CF) has extended their life span. Information about the composition and dynamics of each patient’s microbial community aids in the selection of appropriate treatment of pulmonary exacerbations. We propose the cystic fibrosis rapid response (CFRR) as a fast approach to determine viral and microbial community composition and activity during CF pulmonary exacerbations. The CFRR potential is illustrated with a case study in which a cystic fibrosis fatal exacerbation was characterized by the presence of shigatoxigenic Escherichia coli. The incorporation of the CFRR within the CF clinic could increase the life span and quality of life of CF patients.

## INTRODUCTION

Cystic fibrosis (CF) is a recessive genetic disease in which defects or deficits in the cystic fibrosis transmembrane conductance regulator (CFTR) protein result in disease phenotypes of the pancreas, sweat glands, and reproductive, respiratory, and digestive systems (1). In the lungs of individuals with CF, mucociliary clearance is impaired, which promotes chronic polymicrobial infections (2). Antibiotic treatments and proper disease management have extended the average life span of CF patients; nevertheless, these polymicrobial lung infections are still the primary cause of morbidity and mortality (3). Common bacteria that colonize CF lungs over the long-term include Pseudomonas aeruginosa, Staphylococcus aureus, Haemophilus influenzae, Burkholderia cepacia complex, Rothia mucilaginosa, and *Streptococcus* spp. (4–7), but every CF individual presents a unique microbial community that changes over time (8–10). This highlights the need to characterize the microbial communities in each CF individual.

Microbial community dynamics in CF lungs follow the climax attack model (CAM) (11, 12), in which a climax community is acclimated to the host and dominates during stable periods and a transient attack community is associated with exacerbations. Attack communities are virulent and either colonize the CF lungs from an external source or are already present in the CF lungs and become active during exacerbations. In the CAM, attack communities lead to cystic fibrosis pulmonary exacerbations (CFPEs), declines in lung function, and eventually death. Preventing CFPE relies on quickly identifying attack viral and microbial communities and the genes that they carry and express, such as those encoding specific toxins (13), to efficiently tailor antimicrobial therapies.

Here we propose the cystic fibrosis rapid response (CFRR), a strategy for determining microbial dynamics during CFPE. This strategy is a personalized multi-omics approach that uses viromes (14), metagenomes, metatranscriptomes (15), and metabolomes (7, 16) from longitudinal samples to monitor the whole microbial community, particularly its active members and their metabolic products. Using the CFRR to obtain personalized taxonomic and functional profiles of the lung microbial communities would provide clinicians with comprehensive information about each patient’s viral and microbial ecosystem. This information allows clinicians to generate testable hypotheses, test those hypotheses using standard clinical tests, and propose specific clinical interventions (e.g., precisely targeted antibiotic therapy) to improve CFPE outcomes.

The ability to generate multi-omic data sets and analyze large amounts of data in a clinically relevant time frame (i.e., ≤48 h) makes the CFRR approach applicable in CF clinical practice, especially in clinics closely related to research institutions. It requires access to a sequencing instrument, a mass spectrometer, computational resources, and specialized personnel in each one of these areas. In an optimal situation, the time between sample collection and data interpretation is 30 h for metabolomes (17), 38 h for metagenomes and metatranscriptomes, and 48 h for viromes. These times are expected to shorten as technologies improve. The rapid decrease in sequencing costs (18) and incorporation of sequencing cores within hospitals (19) will increase CF patients’ accessibility to the CFRR in the foreseeable future.

A case study is presented to demonstrate the potential of the CFRR strategy. A 37-year-old male CF patient (CF01) was monitored over a 2-year period with metagenomes, metatranscriptomes, and metabolomes. Integrating the information from these sources led to the identification of an attack community in which a strain of Escherichia coli that likely produced Shiga toxin was detected during a fatal exacerbation.

## RESULTS

### Patient CF01 fatal exacerbation expedited monitoring: metatranscriptomes and metabolomes.

**FIG 1:**
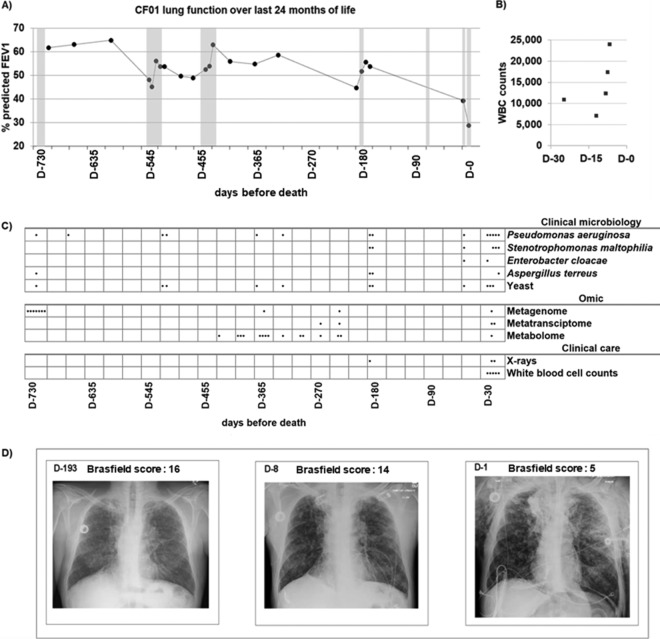
Clinical data for the last 24 months of patient CF01’s life. (A) Percentage of predicted FEV_1_ of patient CF01 over the last 24 months of life. Solid dots are FEV_1_ measurements. The line is included to highlight lung function dynamics and does not represent measurements. Seven exacerbation periods were reported and are shown in gray. (B) White blood cell (WBC) counts for the last month of life. (C) Clinical microbiology positive cultures from patient CF01’s sputum samples over the last 24 months of life. Dots represent days where cultures were positive for each microbe tested in the clinical microbiology panel. Omics sampling points for metagenomes, metatranscriptomes, and metabolomes are indicated by dots in the Omic panel. Performed X rays and WBC measurement days are indicated with dots in the clinical care panel. (D) Patient CF01 chest X rays in a frontal view with quantitative disease severity evaluation using Brasfield scores (74). D-193, mild exacerbation; D-8, acute exacerbation, the time point where CFRR data were obtained; D-1, 1 day before death. A lower Brasfield score represents a higher disease severity. The Brasfield score scale is from 25 to 0, where 25 is lower disease severity and 0 is higher disease severity. Parameters used for Brasfield scores calculations are air trapping, linear markings, nodular cystic lesion, large lesions, and general severity, and individual scores are shown in Table S1A in the supplemental material.

An overall decline in lung function was observed in patient CF01 during his last year of life, and four CFPEs were reported. In the last month of life, 10% of the predicted median forced expiratory volume in 1 s (FEV_1_) was lost (Fig. 1A). During the last exacerbation, the patient was hospitalized at the intensive care unit (ICU) for 7 days and then died. The fatal exacerbation was characterized by severe lung tissue damage (Fig. 1D; see also Table S1A in the supplemental material), an increase in white blood cell counts (Fig. 1B and Table S1B), and a general decline in health. During the fatal exacerbation, clinical microbiology laboratory cultures from sputum samples tested positive for P. aeruginosa, Stenotrophomonas maltophilia, Aspergillus terreus, and yeast (Fig. 1C and Table S1C). Treatment alternated between the antibiotics aztreonam and azithromycin, in addition to a sulfonamide and a quinolone; at the ICU, colistin and meropenem were administered (Table S1D), but no improvement was observed.

10.1128/mBio.00431-19.8 TABLE S1 (A) Brasfield scores of patient CF01’s X rays. (B) Hematology of patient CF01 during his last month of life. (C) Bacterial and fungal cell culture results from the clinical microbiology laboratory for patient CF01 during his last 2 years of life. (D) Antibiotic received as treatment during the last 2 years of patient CF01’s life. (E) Metagenome and metatranscriptome sequencing overview for patient CF01’s sputum samples. Download Table S1, DOCX file, 0.06 MB. Copyright © 2019 Cobián Güemes et al. 2019 Cobián Güemes et al. This content is distributed under the terms of the Creative Commons Attribution 4.0 International license.

The CFRR strategy was launched to rapidly identify the cause of the CFPE. Sputum samples were collected 7 and 8 days before death (samples D-7 and D-8). In samples D-7 and D-8, active members of the microbial community were determined using metatranscriptomics. In sample D-8, small-molecule profiles (using metabolomics) were characterized, and a total DNA metagenome was sequenced.

**FIG 2:**
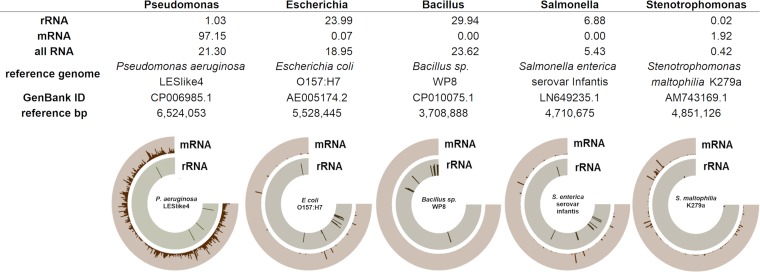
The most abundant bacterial genera of fatal exacerbation sample D-8. The relative abundances of each genus as determined by rRNA, mRNA, and total RNA are shown. A reference genome from each genus was selected based on the number of reads recruited in the rRNA (*Escherichia*, *Bacillus*, and *Salmonella*) or mRNA (*Pseudomonas* and *Stenotrophomonas*) category. Fragment recruitment was visualized using Anvi’o, showing a logarithmic scale for mRNA and rRNA from 1 to 1,000. Anvi’o plots show reads mapped along the genome coordinates. Nonribosomal microbial reads were recruited against each reference genome using SMALT with an identity cutoff of 80% and are shown in brown along the external ring. rRNA reads were classified into each genus by BLASTn, were recruited against the corresponding reference genome using SMALT with an identity cutoff of 60%, and are shown in gray along the internal ring.

Metatranscriptomics data from sample D-8 showed that the most abundant microbial rRNAs belonged to the genera *Bacillus* (29.9%), *Escherichia-Shigella* (23.9%), *Streptococcus* (11.6%), *Salmonella* (6.9%), and *Lactococcus* (4.4%), among other genera (23.3%) (Fig. S1A). The microbial mRNA composition was dominated by the genus *Pseudomonas* (97.1%), followed by *Stenotrophomonas* (1.9%) and *Escherichia* (0.07%) (Fig. S1B). At species-level resolution, the most abundant bacterial genomes (based on total RNA) (Fig. 2) were *Bacillus* sp., shigatoxigenic E. coli (STEC), Salmonella enterica serovar Infantis, P. aeruginosa, and S. maltophilia. Enterobacterial phage SP6, *Pseudomonas* phages, and *Stenotrophomonas* phage S1 were also detected. Two members of the phylum Ascomycota were identified: Candida albicans and Aspergillus fumigatus. Metagenomics data of sample D-8 identified *Pseudomonas* (98.5%) as the dominant bacterial genus (Fig. S5A).

10.1128/mBio.00431-19.1 FIG S1 Total bacterial RNA composition during a fatal exacerbation (sample D-8). Download FIG S1, PDF file, 0.2 MB. Copyright © 2019 Cobián Güemes et al. 2019 Cobián Güemes et al. This content is distributed under the terms of the Creative Commons Attribution 4.0 International license.

**FIG 3:**
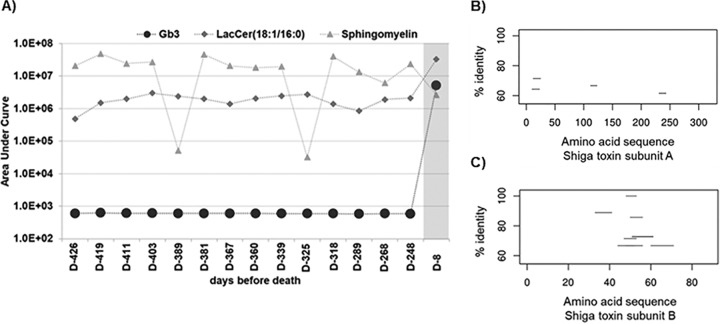
Shiga toxin and its human receptor globotriaosylceramide (Gb3). (A) The masses of globotriaosylceramide and its precursors lactosylceramide and sphingomyelin from exacerbation sample D-8 and 14 historical nonexacerbation samples were determined by parent mass searching and validated by MS/MS matching. The fatal exacerbation sample is shown in gray. (B) STEC BRF1 was used as a reference genome for fragment recruitment to the Shiga-like toxin 2 subunit A protein sequence. The amino acid sequence position is shown on the *x* axis, and percent identity is shown on the *y* axis. The nucleotide sequences from patient CF01 metatranscriptome exacerbation sample D-8 were mapped to proteins using BLASTx with an E value cutoff of 0.001 and filtered by an identity of >60%. (C) Metatranscriptome recruitment as explained above for panel B, except that in this case, reads were recruited to the Shiga-like toxin 2 subunit B amino acid sequence.

The presence of *Escherichia-Shigella* in the lungs of a CF patient is unusual, and thus, a detailed analysis was performed to further resolve the taxonomy at the strain level. Strain-level analysis identified that E. coli present in patient CF01’s lungs was most closely related to the genome of E. coli (STEC) B2F1. This strain typically carries the Shiga toxin 1 and Shiga toxin 2 genes, both of which were identified in the metatranscriptomes (Fig. 3B and C). Furthermore, the Shiga toxin receptor globotriaosylceramide (Gb3) was detected in the metabolome from sample D-8 (Fig. 3A). This suggests that Shiga toxin and its Gb3 target were being produced in the lungs of patient CF01. Gb3 is produced in human cells by Gb3 synthase, which adds a sugar to a lactosylceramide molecule. Ceramide is produced by sphingomyelinase (SMase) in the host cell or by the action of bacterially encoded SMase (see Fig. 5B). The gene that encodes a P. aeruginosa secreted SMase, the hemolytic phospholipase C (PlcH) (20), was detected in the sample D-8 metatranscriptome (Fig. S2B).

10.1128/mBio.00431-19.2 FIG S2 (A) P. aeruginosa gene expression during a stable period (samples D-279 and D-303) and fatal exacerbation (samples D-7 and D-8) based on fragment recruitment to the P. aeruginosa PAO1 reference genome. (B) P. aeruginosa SMase *plcH* coverage plot. (C) Predicted prophage 1 from the assembled genome of P. aeruginosa CF01. (D) Predicted prophage 2 from the assembled genome of P. aeruginosa CF0. Download FIG S2, PDF file, 0.2 MB. Copyright © 2019 Cobián Güemes et al. 2019 Cobián Güemes et al. This content is distributed under the terms of the Creative Commons Attribution 4.0 International license.

In a longitudinal metabolomics data set, Gb3 was highly abundant (*P* < 0.0001) in sample D-8 but was in low abundance in the prior samples (Fig. 3A). The Gb3 precursor lactosylceramide (18:1/16:0) (21) and its ceramide donor sphingomyelin (18:1/16:0) (22) were abundant in all samples throughout the longitudinal data set (Fig. 3A and Table S2A). These data demonstrate that Gb3 precursors were present for at least a year before the fatal exacerbation, but Gb3 was produced in significantly high quantities 8 days before death (sample D-8).

10.1128/mBio.00431-19.9 TABLE S2 (A) Comparison of molecule spectra between nonexacerbation samples (samples D-426 to D-248) and exacerbation sample D-8. (B) Comparison of numbers of specific bacterial spectra between nonexacerbation samples (samples D-426 to D-248) and exacerbation sample D-8. Download Table S2, DOCX file, 0.05 MB. Copyright © 2019 Cobián Güemes et al. 2019 Cobián Güemes et al. This content is distributed under the terms of the Creative Commons Attribution 4.0 International license.

Gb3 levels positively correlate with Shiga toxin levels (23), although the mechanism behind this positive correlation is not clear. Gb3 is the only known functional receptor for Shiga toxins (24), and Shiga toxins induce reorganization of lipids in the epithelial cell’s membrane. Shiga toxin B can bind up to 15 Gb3 molecules (25), and this binding results in the aggregation of Gb3 in lipid rafts. The aggregation of Gb3 in lipid rafts promotes a negative membrane curvature and internalization of Shiga toxin (26). The spatial distribution of Gb3 in the cell membrane has a regulatory role in its presentation (27); thus, higher recruitment of Gb3 in lipid rafts may induce the production of more Gb3.

Antibiotic resistance genes were detected in the metatranscriptomes of samples D-8 and D-7. Transcripts encoding all the protein components were identified for two RND-type multidrug exporters, MexGHI-OpmD (28) and MexA-MexB-OprM (29), previously described in *Pseudomonas*, as well as the tetracycline efflux pump Tet(C), previously described in *Achromobacter*. Transcripts encoding several beta-lactamases were identified, such as TEM-116, PDC-3, OXA-50, and BEL-3 (30), which are typically found in *Pseudomonas*, and CTX-M-21 (31), which is usually found in *Enterobacteriaceae*. Transcripts encoding enzymes that are involved in resistance to macrolide, aminoglycoside, lincosamide, diaminopyrimidine, and glycopeptide antibiotics were detected; these enzymes were previously described in *Pseudomonas*, *Achromobacter*, *Escherichia*, *Streptomyces*, *Paenibacillus*, *Clostridium*, and *Morganella* (Table S3A).

10.1128/mBio.00431-19.10 TABLE S3 (A) Antibiotic resistance genes present in exacerbation metatranscriptomes. (B) Genes that are predicted to encode resistance to antibiotics and that were present in *Pseudomonas* contigs assembled from metatranscriptome reads sampled during the exacerbation. Download Table S3, DOCX file, 0.06 MB. Copyright © 2019 Cobián Güemes et al. 2019 Cobián Güemes et al. This content is distributed under the terms of the Creative Commons Attribution 4.0 International license.

A partial P. aeruginosa genome sequence was recovered by assembling reads from the fatal exacerbation metatranscriptomes (samples D-8 and D-7) into contigs and then mapping those contigs to the P. aeruginosa PAO1 reference genome (Fig. S2A). In the resulting P. aeruginosa CF01 contigs, 38 genes related to resistance to antibiotics and toxic compounds were identified (Table S3B). Two prophages were also identified in the assembled P. aeruginosa CF01 contigs (samples D-8 and D-7); one was complete, and the second one was a partial prophage (Fig. S2C and D).

### Bacterial small-molecule profiles before and during fatal exacerbation.

Longitudinal metabolomic data from patient CF01’s historical samples and fatal exacerbation sample D-8 were compared to metabolic profiles from six pathogenic bacterial isolates previously detected in CF sputum (P. aeruginosa VVP172, *Enterococcus* sp. strain VVP100, E. coli VVP427, *Streptococcus* sp. strain VVP047, *Stenotrophomonas* sp. strain VVP327, and S. aureus VVP270). The goal was to identify metabolites produced by pathogenic bacteria and track how changes in their abundances might have preceded the fatal exacerbation. Metabolites from these pathogens were consistently detected throughout the longitudinal samples. In sample D-8, there was an increase (*P* < 0.001) in the number of metabolites that matched P. aeruginosa VVP172, E. coli VVP427, *Streptococcus* sp. VVP047, and S. aureus VVP270 (Fig. S3 and Table S2B).

10.1128/mBio.00431-19.3 FIG S3 Metabolomes from sample D-8 and their comparison to historical samples for patient CF01. Download FIG S3, PDF file, 0.09 MB. Copyright © 2019 Cobián Güemes et al. 2019 Cobián Güemes et al. This content is distributed under the terms of the Creative Commons Attribution 4.0 International license.

### Active members of the microbial community during a stable period and the fatal exacerbation.

**FIG 4:**
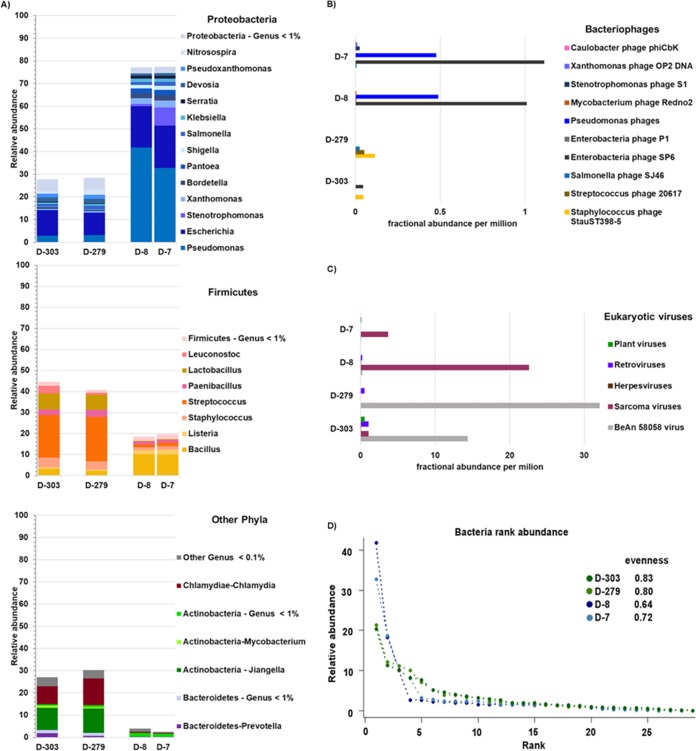
Actively transcribing members of the viral and bacterial communities in sputum samples of patient CF01. Metatranscriptomes from two exacerbations and two stable samples were obtained. (A) Bacterial taxonomical assignments were made using KAIJU at the genus level and are color-coded by phylum. (B) Fractional abundances of bacteriophages based on viral RefSeq mapping and FRAP normalization. (C) Fractional abundances of eukaryotic viruses based on viral RefSeq mapping and FRAP normalization. (D) Bacterial rank abundance plot, generated using relative abundances at the genus level. Evenness was calculated as *H*/In(*S*), where *H* is the Shannon diversity index and *S* is the total number of species.

Analysis of metatranscriptomes from a stable period 10 and 9 months before the fatal exacerbation event (samples D-303 and D-279) identified several differences between this stable period and the fatal exacerbation. First, the phylum *Firmicutes* was the most active phylum during the stable period, whereas the phylum *Proteobacteria* was the most active during exacerbation (Fig. 4A). Second, samples from the stable period showed an active microbial community that was more even and diverse than the community in exacerbation samples (Fig. 4D). Third, transcripts from *Pseudomonas* were detected at very low levels in stable samples (average relative abundance, 3%) but at high levels in exacerbation samples (average relative abundance, 37%) (Fig. S2A). Fourth, the percentages of unclassified sequences were higher in stable samples D-303 and D-279 (40.9% and 39.0%) than in exacerbation samples D-8 and D-7 (27.6% and 17.0%). Fifth, a higher fractional abundance of bacteriophages was detected in the fatal exacerbation samples than in the stable ones. Enterobacterial phage SP6, several *Pseudomonas* phages (Fig. 4B), and sarcoma viruses (Fig. 4C) were the dominant viruses in samples D-8 and D-7.

### Microbial community dynamics during a nonfatal exacerbation.

Two years before the fatal exacerbation, patient CF01’s lung function declined faster than in previous years (Fig. S4A). The rate of lung function change in the last 2 years of life was −9.75 FEV_1_%/year (Fig. S4C). The overall rate of lung function change during patient CF01’s last 14 years of life was −1.39 FEV_1_%/year. During a 2-year period of 4 and 3 years before death, the rate of lung function change was 1.30 FEV_1_%/year (Fig. S4B).

10.1128/mBio.00431-19.4 FIG S4 (A) Percentage of predicted FEV_1_ of patient CF01 for 14 years. (B) Percentage of predicted FEV_1_ of patient CF01 for years 4 and 3 before death. (C) Percentage of predicted FEV_1_ of patient CF01 for the last 2 years of life. Download FIG S4, PDF file, 0.1 MB. Copyright © 2019 Cobián Güemes et al. 2019 Cobián Güemes et al. This content is distributed under the terms of the Creative Commons Attribution 4.0 International license.

During the 2-year period leading up to the fatal exacerbation, seven exacerbation events were reported, and sputum samples were periodically screened for fungi and bacteria at the clinical microbiology laboratory (Table S1C). P. aeruginosa was detected in all samples. Six months before the fatal exacerbation, S. maltophilia was detected, and during the last 2 months of life, Enterobacter cloacae was detected. A. terreus was detected in two samples in the last 6 months of life. Yeast was detected in all screened samples, except for the final exacerbation samples. Based on this information, several antibiotics were prescribed to manage the exacerbations (Table S1D); these included monobactams, macrolides, quinolones, beta-lactams, sulfonamides, and a cationic polypeptide.

Two years before patient CF01’s death, metagenomics was used to monitor the microbial composition of the respiratory tract during an exacerbation event, the subsequent antibiotic treatment (samples D-724 to D-718), and a stable period that followed (samples D-409 and D-286) (Fig. S5A). The bacterial genera that best differentiated between samples collected during periods of antibiotic treatment (D-722 to D-718) and no antibiotic treatment (D-724 and D-723) were *Rothia*, *Campylobacter*, *Veillonella*, and *Prevotella* (Fig. S6). The antibiotics prescribed during this exacerbation were a fluoroquinolone (ciprofloxacin) and a tetracycline (doxycycline). Clinical microbiology laboratory tests performed on sample D-719 were positive for P. aeruginosa, Pseudomonas fluorescens, A. fumigatus, and yeast (Table S1C). Exacerbation and stable samples had *Streptococcus* phages, *Staphylococcus* phages, and *Pseudomonas* phages, whereas only exacerbation samples had a Shiga toxin-converting phage (Fig. S5B), and stable samples had higher abundances of herpesviruses (Fig. S5D).

10.1128/mBio.00431-19.5 FIG S5 Metagenomic analysis was performed on sputum samples collected over a 7-day exacerbation period, during a subsequent stable period of 10 to 14 months, and during fatal exacerbation. Download FIG S5, PDF file, 0.3 MB. Copyright © 2019 Cobián Güemes et al. 2019 Cobián Güemes et al. This content is distributed under the terms of the Creative Commons Attribution 4.0 International license.

10.1128/mBio.00431-19.6 FIG S6 Variable-importance plot using mean decrease accuracy for a supervised random forest with 5,000 trees. Download FIG S6, PDF file, 0.1 MB. Copyright © 2019 Cobián Güemes et al. 2019 Cobián Güemes et al. This content is distributed under the terms of the Creative Commons Attribution 4.0 International license.

## DISCUSSION

The unusually fast decline of patient CF01 led to the implementation of the CFRR. During patient CF01’s fatal exacerbation, E. coli mRNA, rRNA, and metabolites were detected, which demonstrated not only the presence but also the activity of shigatoxigenic E. coli. The identification of a shigatoxigenic E. coli strain is supported by rRNA (36,590 unique rRNA sequences in metatranscriptome D-8), mRNA (1,412 E. coli mRNA reads in metatranscriptome D-8 and 11 partial mRNA reads with 60% identity to STEC BRF1), and metabolites (10 metabolome spectra matched to E. coli in sample D-8). The presence of STEC in the lungs of a CF patient was alarming, as this strain causes severe damage to the lung epithelium (32, 33). Moreover, interactions between Shiga toxin and the host epithelium were inferred from metabolomes. The molecule Gb3, the receptor for Shiga toxin, showed an increase of 3 orders of magnitude during the fatal exacerbation (sample D-8), compared to previous samples.

**FIG 5:**
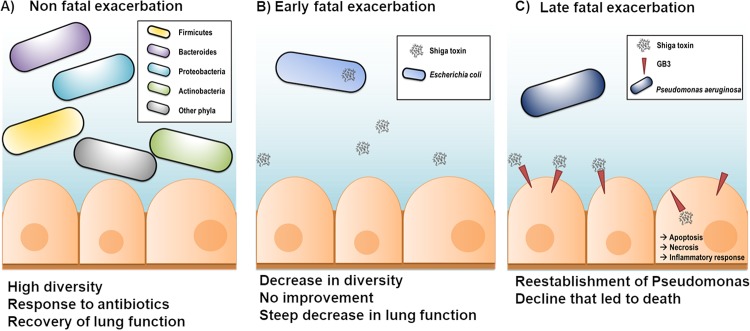
Proposed model of lung dynamics resulting in patient CF01’s death. (A) A nonfatal exacerbation (days −724 to −718) was followed by a recovery of lung function, and attack and climax communities were diverse. (B) The fatal exacerbation was triggered by colonization by STEC, which is supported by the presence of its rRNA in the metatranscriptomes. This bacterium encodes Shiga toxin, which was likely taken into host cells by the human receptor globotriaosylceramide. (C) Later during the fatal exacerbation, Shiga toxin was internalized and then induced apoptosis, necrosis, and inflammation. P. aeruginosa was reestablished and came to dominate the community, as suggested by its abundant mRNA.

Altogether, these multi-omics data support the following model of microbial dynamics that caused patient CF01’s death. At the beginning of the fatal exacerbation, STEC produced Shiga toxin that remained inside the bacterial cells. Later in the exacerbation, STEC’s cell membranes were disrupted, and the Shiga toxin was released (Fig. 5B). This release may have been triggered by the action of the cationic polypeptide colistin (34). Next, the toxin was taken up by lung epithelial cells through the host membrane receptor globotriaosylceramide (35) (Fig. 5C). Inside the lung epithelial cells (32), Shiga toxin inhibited host translation by blocking the ribosomes, thereby inducing cell death, necrosis, and an acute inflammatory response (32, 36, 37). The immune response and lung tissue damage were evident in the chest X rays and the increase in white blood cells (samples D-8 and D-1) (Fig. 1D).

During the fatal exacerbation, STEC led the attack community that ultimately destabilized the climax community, a phenomenon previously reported in CF exacerbations (11); this resulted in declines of evenness (diversity index that quantifies how equal the community is [38]) and diversity (the number of different species in a community [39]), a switch from a community dominated by *Firmicutes* to one dominated by *Proteobacteria*, and transcription of enterobacterial and *Pseudomonas* bacteriophages and sarcoma viruses. This event was followed by a *Pseudomonas* and *Stenotrophomonas* bloom, characterized by active transcription, as both rRNA and mRNA were detected, as was an increase in their metabolites. *Pseudomonas* was the most active member of the microbial community, with an mRNA abundance of 97%, followed by 1.92% for *Stenotrophomonas* mRNA. *Bacillus* was either lysed or dormant, as only rRNA was detected. A feature that may have contributed to the success of *Pseudomonas* was its resistance to multiple antibiotics, as detected by the transcription of over 38 antibiotic resistance genes. This scenario is congruent with the one described by the clinical laboratory, as cultures positive for *Pseudomonas* and *Stenotrophomonas* were reported during the fatal exacerbation.

Additional dynamics such as bacteriophage induction may have happened during the fatal exacerbation, as active transcription was detected from enterobacterial phage SP6 and *Pseudomonas* bacteriophages. Bacteriophage induction is known to play a role in the control of bacterial populations in CF lungs (40).

### CFRR for polymicrobial infection management, the importance of historical samples, and a fast sample-to-result strategy.

The CFRR emerged from the need to investigate the cause of acute exacerbations. The power of the CFRR is shown in the information obtained for the patient CF01 case study. The CFRR is ideal for medical centers closely associated with research facilities where the equipment is available. However, as technologies improve and become more accessible, the CFRR could be implemented within the clinic.

A key component of the CFRR strategy is the comparison between acute exacerbations and stable periods. Because CF microbial communities are heterogeneous, a baseline needs to be determined for each patient. Longitudinal samples are essential to identify the changes in the microbial community and metabolites during acute exacerbations.

In the presented patient CF01 case study, historical samples were essential to differentiate the attack community that led to a fatal exacerbation from the attack community associated with a nonfatal exacerbation. The increase in Gb3 abundance during patient CF01’s fatal exacerbation (Fig. 3) was detected by comparing its abundances in historical samples. In the case of metabolites, a baseline is necessary for each CF patient because for many compounds, the basal levels are not known. Accumulation of ceramides and sphingomyelins is observed in CF lungs (41). In particular, levels of sphingomyelins, ceramides, and lactosylceramide are significantly higher in CF lungs than in non-CF ones (42).

A challenging component of the CFRR is the collection and storage of historical samples. Sputum samples intended for virome, metagenome, and metabolome (43) analyses are stable if stored at −20°C or −80°C. Metatranscriptomes are prone to RNA degradation, and sputum collection intended for this purpose requires RNA stabilization prior to −20°C or −80°C storage. Given these considerations, each patient can be provided with a non-thaw-cycle −20°C freezer where individual raw sputum samples can be stored for viromes, metagenomes, and metabolomes (see Fig. S7A in the supplemental material). Sputum samples for metatranscriptomes can be collected during the patient’s visit to the CF clinic, where immediately after collection, the RNA integrity is preserved by adding TRIzol or RNAlater. RNA should then be extracted as soon as possible. A sampling scheme in which a higher resolution of samples is desired close to an acute exacerbation and fewer samples are desired far away from the exacerbation event is proposed (Fig. S7).

10.1128/mBio.00431-19.7 FIG S7 Sampling scheme for collection of “historical” sputum samples. Download FIG S7, PDF file, 0.2 MB. Copyright © 2019 Cobián Güemes et al. 2019 Cobián Güemes et al. This content is distributed under the terms of the Creative Commons Attribution 4.0 International license.

**FIG 6:**
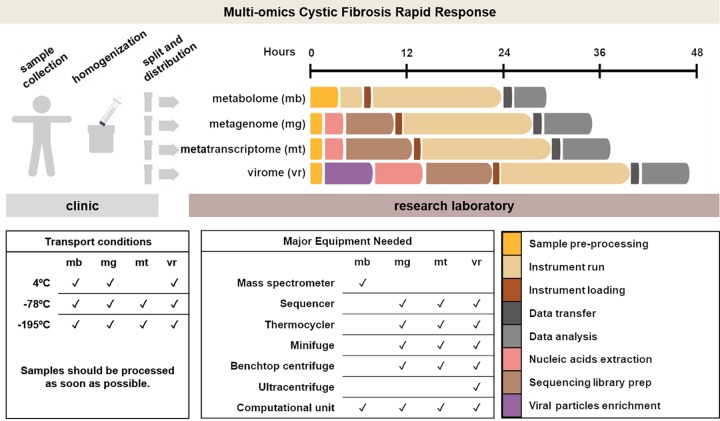
Cystic fibrosis rapid response. Our proposed multi-omics strategy is to analyze sputum samples from cystic fibrosis patients, in which metabolomes, metagenomes, metatranscriptomes, and viromes are obtained from a single sputum sample. Estimated times and equipment for each omics step are included, as are recommended transport conditions. Recommended transport condition temperatures can be achieved by using ice, dry ice, or liquid nitrogen.

Historical samples collected by the patient at home or during routine visits to the clinic are a valuable resource in the event of an acute exacerbation. In these cases, historical samples would be processed along with those from acute exacerbations in the CFRR pipeline (Fig. 6), and valuable information would be obtained in less than 48 h. This information is then analyzed by a multidisciplinary scientific team along with the clinician to (i) validate the multi-omics findings with approved clinical tests and (ii) identify appropriate therapeutic options.

The information presented by the CFRR to the clinician is more detailed than that provided by classical clinical microbiology. A clear understanding of how this information is obtained and the exploratory nature of the findings needs to be considered when interpreting the results. Discussion among clinicians and experts on the benefits and limitations of each omics approach is essential to identify the elements causing CF acute exacerbations and then select the course of action to prevent a fatality. The final treatment decision is always in the hands of the clinician, who evaluates the different lines of evidence for each finding and considers the cost-to-benefit ratio of possible therapeutic interventions. The application of the CFRR in a clinical context gives CF patients the opportunity for a better outcome based on an informed treatment decision. Another consideration when implementing the CFRR in the clinic is the availability of financial resources to perform the multi-omics strategy on exacerbation and historical samples.

### Considerations about implementing the cystic fibrosis rapid response.

This was a retrospective study in which the patient’s treatment was not modified based on the presented meta-omics results. The course of action of the CFRR strategy is to provide information to clinicians so that they can evaluate and confirm the findings before proceeding with pertinent treatment modifications.

In the case of patient CF01’s fatal exacerbation, the information obtained from the CFRR strategy could have informed the course of action of the treatment with the following modifications: (i) use of different antibiotics, since the mechanism of action of colistin results in liberation of the bacterial cell contents, such as Shiga toxin, and (ii) administration of neutralizing antibodies against Shiga toxin. Colistin is a cationic polypeptide that disrupts the cell membrane of Gram-negative bacteria through a detergent-like mechanism, and it is often used in the treatment of multidrug-resistant exacerbation in patients with CF (44).

In the presented case study, only metatranscriptomes, metabolomes, and metagenomes were used to elucidate the cause of a fatal exacerbation. In future CFRR case studies, the use of viromes could be incorporated. The combination of metagenomes and viromes allows the identification of viral induction events, for example, of prophages carrying toxins. Shigatoxigenic phages are capable of lysogenic conversion (45, 46), and in the case of patient CF01’s fatal exacerbation, an early detection of Shiga toxin in the viromes of historical samples could have provided valuable information about the coding potential of the viral community.

Time is crucial during the management of CF exacerbations. The estimated execution time of the CFRR in an ideal situation with specialized staff working 24/7 is 48 h. Each step has room for improvement that would shorten the execution times. For example, real-time direct sequencing, such as Oxford Nanopore, can eventually be used for CFRR metagenomes, metatranscriptomes, and viromes. These technologies provide genomic information as it is being sequenced (47, 48), which will be ideal for the CFRR once sample preparation and data analysis are optimized for human DNA removal (49) and once large amounts of sputum starting material (400 ng of DNA needed for a Nanopore run) are no longer necessary for DNA sequencing.

Combining data from multiple omics sources enabled the identification of shigatoxigenic E. coli as the likely cause of patient CF01’s fatal exacerbation. Although these omics data were not used to alter clinical treatment of patient CF01, future applications of the CFRR are expected to provide information that is essential for improving therapy, e.g., antibiotic resistance predictions and gene expression in major attack community pathogens. Although each individual’s CF community is unique, these methods will allow for the observation of overarching trends within and between patients, for example, a loss in diversity in acute exacerbations.

## MATERIALS AND METHODS

### Clinical data.

Sample collection procedures and access to clinical data were approved by the institutional review boards (IRBs) of the University of California San Diego (UCSD) (HRPP 081510), and San Diego State University (IRB approval number 1711018R). Clinical microbiology, hematology, and X rays were performed during the normal care of the patient at the UCSD medical center. Spirometry tests were used to calculate the percentage of predicted FEV_1_ as previously described (50). Clinical status (exacerbation or stable) was determined by the clinician. Lung function dynamics were modeled using splines and linear model fitting as previously described (51).

### Metagenome and metatranscriptome shotgun sequencing.

Sputum samples were collected by expectoration in a sterile cup and processed for metagenomes or metatranscriptomes as previously described (52). Metagenome libraries were constructed using a Nextera DNA library preparation kit. Metatranscriptome libraries were constructed using a TruSeq RNA library preparation kit. All libraries were sequenced on the Illumina GAIIx platform. Metatranscriptomes D-7 and D-8 were prepared using a modified procedure to obtain rRNA and mRNA in a single sequencing step, where half of the sample was depleted of rRNA using a Ribo-Zero gold kit (15) and total RNA was extracted from the other half. Both fractions were pooled in a proportion of 4:1, and a single Illumina library was then constructed and sequenced.

### Sequencing data processing.

Quality filtering and dereplication were done using PRINSEQ (53) (-min_qual_mean 20 -derep 1245 -lc_method entropy -lc_threshold 50 -ns_max_p 1 -out_bad null). Cloning vector sequences were removed using SMALT (-y 0.8 -x) with 80% identity against the UniVec database (54); possible sources of cloning vector sequences are reagents used in the library preparation (55, 56). Human genome sequences were removed using BLASTn (E value of 0.1) against the human reference genome GRCh38. Metagenome and metatranscriptome data sets presented in this study are summarized in Table S1E in the supplemental material. Microbial taxonomy assignments at the genus level were made from BLASTn against the nucleotide (NT) database (E value of 0.001; the hit with the lowest E value out of 10 hits was kept) for metagenomes and KAIJU (57) for metatranscriptomes. Viral assignments were made by mapping reads against the viral reference genome database (NCBI RefSeq, release 87) using SMALT (58) with 80% identity. Fractional abundances were calculated using FRAP as previously described (59) and expressed per million reads. After quality filtering and removal of reads that mapped to the human genome, metatranscriptome D-8 reads were compared to the SILVA SSU database using BLASTn with an E value cutoff of 0.001, and taxonomy was assigned at the genus level using the best hit from 10,000 subsample replicates. Nonribosomal reads were compared to the NCBI NT database using BLASTn with an E value cutoff of 0.001. The best hit was selected and used to assign bacterial taxonomy at the genus level. Species-level assignments were determined by the genome that recruited the most reads for each genus at either the rRNA (*Bacillus*, *Escherichia*, and *Salmonella*) or mRNA (*Pseudomonas* and *Stenotrophomonas*) level. The bacterial genome with more hits in the BLASTn analysis was selected as the closest strain and used as the reference genome. rRNA and mRNA reads were mapped against each one of the reference genomes using SMALT with identity cutoffs of 60% and 80%, respectively, and the results were visualized using Anvi’o (60).

Reads from metatranscriptomes D-8 and D-7 were together assembled *de novo* using SPADES (61), and all resultant contigs were compared to the NT database using BLASTn with an E value cutoff of 0.001; taxonomies were assigned using MEGAN6 (62). Contigs identified as *Pseudomonas* in all metatranscriptomes were separately mapped to the reference genome of P. aeruginosa PAO1 using SMALT with an identity cutoff of 80%. *Pseudomonas* contigs (*n* = 4965; total of 2,686,355 bp) were annotated using PATRIC (63); genes identified by subsystem classification as encoding resistance to antibiotics and toxic compounds are summarized in Table S3A in the supplemental material. All contigs were screened for antibiotic resistance genes using the Resistance Gene Identifier implemented in the CARD database (30). All perfect and strict hits were retained, as was any hit with an identity of ≥80%. Metatranscriptome D-8 and D-7 reads were mapped to the proteins Shiga-like toxin subunit A and subunit B using BLASTx with an E value cutoff of 0.001 and an identity of 60%. Fragment recruitment plots were generated using custom python scripts.

### Sample comparison.

Random forest, a nonparametric statistical method, was used to determine the bacterial genera that best differentiated between (i) antibiotic treatment and no antibiotic treatment in the metagenomes and (ii) stable and exacerbation states in the metatranscriptomes. The importance of each variable was assessed using the R implementation of the algorithm random forest (64), using 2,000 trees.

The R package vegan (65) was used with the metatranscriptomes to calculate Pielou’s evenness using Shannon diversity.

### Metabolomics.

Liquid chromatography-tandem mass spectrometry (LC-MS/MS) metabolomics data were generated from sputum sample D-8 and compared to those of a set of 15 samples routinely collected from the previous 426 days. Metabolite extraction (ethyl acetate and methanol), LC-MS/MS methods, and data analysis were performed as described previously (16). Data from these same sputum samples have been reported previously (16), but the metabolites reported here were not presented in that study, making these data novel (MassiVE data set MSV000079444).

### Metabolomics data processing.

Metabolomics data were analyzed using molecular networking (66) and Global Natural Products Social Molecular Networking (GNPS) (67). Molecular networking parameters were altered for this study and are as follows: cosine minimum of 0.7, 6 minimum matched peaks for spectral clustering, and precursor mass and fragment ion mass tolerance of 0.1 Da. Molecular networks were visualized using Cytoscape software (68). Molecules were annotated by searching the GNPS libraries, and specific metabolites of interest were searched for using the MS^1^ parent mass and then compared to the Metlin MS/MS spectral libraries (69). Area under the curve abundances of metabolites in the LC-MS/MS data were calculated using mzMine 2 software (70), using selected masses. The parameters of the feature finding were as follows: minimum time span of 0.05 min, minimum feature height of 2, and *m/z* tolerance of 0.05 *m/z* or 15.0 ppm. The chromatograms were deconvoluted, isotope peaks were grouped, and the peaks were aligned with the same ion mass tolerance and a retention time tolerance of 1 min. The final matrix of features was gap filled. All metabolite annotations based on spectral alignment are considered level 2 according to proposed minimum reporting standards for metabolomics (71).

Isolates of CF pathogens P. aeruginosa VVP172, *Enterococcus* sp. VVP100, Escherichia coli VVP427, *Streptococcus* sp. VVP047, Stenotrophomonas maltophilia VVP327, and Staphylococcus aureus VVP270 were obtained from the UCSD Center for Advanced Laboratory Medicine. These isolates were grown in artificial sputum medium according to a method described previously (12), and their metabolomes were extracted using sequential extraction with ethyl acetate and methanol (the same method as for the sputum samples described in reference 16). The LC-MS/MS data were generated with the same protocols as those for the sputum samples, and the data were uploaded to GNPS. The MS/MS data from these bacterial isolates were used individually as a reference for searching for matching spectra in patient CF01’s longitudinal sputum data. Spectral matching parameters were as follows: parent and fragment mass tolerance of 0.1, minimum matched peaks of 6, cosine of 0.7, and minimum spectral count of 3 in the data set. Spectral matches between a sputum sample file and a bacterial isolate were summed for each sample for each bacterium and plotted to identify metabolite matches through the longitudinal data sets from pathogens known to be present in patient CF01 from clinical culture history (it must be noted these isolates were obtained from CF patients in the same clinic as patient CF01 but not from patient CF01). It is unknown if specific bacterial molecules were detected.

### Data availability.

Sequencing data are available at the SRA under accession number SRP173673 (72). Metabolomics data are available on GNPS with MassiVE data set MSV000079444 (73). The resulting FASTA files are available in the NCBI Sequence Read Archive (SRA) with the following accession numbers: SAMN10605049 to SAMN10605062 (*n* = 12).
