# Supplementary material for: Cystic Fibrosis Rapid Response: Translating Multi-omics Data into Clinically Relevant Information
Source: mBio. 2019 Apr 16;10(2):e00431-19. doi: 10.1128/mBio.00431-19 (PMC6469968; doi:10.1128/mBio.00431-19)
Supplement: TABLE S1 [file mBio.00431-19-st001.docx]

**Supplemental Table 1A**. Brasfield scores of CF01 X-rays. X-rays were performed as part of the patient’s regular clinical care.

| Day | Air trapping | Linear markings | Nodular cystic lesions | Large lesions | General severity | Brasfield score |
| --- | --- | --- | --- | --- | --- | --- |
| D-1 | 3 | 4 | 4 | 5 | 4 | 5 |
| D-8 | 2 | 3 | 3 | 0 | 3 | 14 |
| D-193 | 2 | 2 | 2 | 0 | 2 | 16 |

**Supplemental Table 1B**. Hematology of CF01 during their last month of life. White blood cell counts are expressed in cells per microliter.

| Day | White Blood Cells count | segmented neutrophils |
| --- | --- | --- |
| D-0 | 24,000 | 96% |
| D-1 | 17,400 | 96% |
| D-2 | 12,400 | 95% |
| D-7 | 7,100 | 97% |
| D-23 | 10,900 | 95% |

**Supplemental Table 1C**. Bacteria and fungi cell culture results from the clinical microbiology laboratory for patient CF01 during their last two years of life.

| **Day** | **Status** | **Bacteria** | **Fungi** |
| --- | --- | --- | --- |
| D-0 | exacerbation | *Pseudomonas aeruginosa* | *Aspergillus terreus* |
|  |  | *Stenotrophomonas maltophilia* |  |
| D-1 | exacerbation | *Pseudomonas aeruginosa* |  |
|  |  | *Stenotrophomonas maltophilia* |  |
| D-7 | exacerbation | *Pseudomonas aeruginosa* | Yeast |
|  |  | *Stenotrophomonas maltophilia* |  |
| D-14 | exacerbation | *Pseudomonas aeruginosa* | Yeast |
| D-24 | exacerbation | *Pseudomonas aeruginosa* | Yeast |
|  |  | *Enterobacter cloacae* |  |
| D-48 | stable | *Pseudomonas aeruginosa* | Yeast |
|  |  | *Stenotrophomonas maltophilia* |  |
|  |  | *Enterobacter cloacae* |  |
| D-192 | exacerbation | *Pseudomonas aeruginosa* | *Aspergillus terreus* |
|  |  | *Stenotrophomonas maltophilia* | Yeast |
| D-204 | exacerbation | *Pseudomonas aeruginosa* | Yeast |
|  |  | *Stenotrophomonas maltophilia* |  |
| D-373 | stable | *Pseudomonas aeruginosa* | Yeast |
| D-414 | stable | *Pseudomonas aeruginosa* | Yeast |
| D-540 | stable | *Pseudomonas aeruginosa* | Yeast |
| D-547 | stable | *Pseudomonas aeruginosa* | Yeast |
| D-674 | stable | *Pseudomonas aeruginosa* |  |
| D-719 | exacerbation | *Pseudomonas aeruginosa* | Yeast |
|  |  | *Pseudomonas fluorescens* | *Aspergillus fumigatus* |

**Supplemental Table 1D.** Antibiotic received as treatment during the last two years of CF01’s life. Class and mechanism of action were obtained from PubChem and DrugBank.

| Month | Antibiotic | Class | Comments | Mechanism of action |
| --- | --- | --- | --- | --- |
| M-0 | Aztreonam | monobactam |  | Inhibit synthesis of bacteria cell wall, binds to and inactivates penicillin-binding-protein-3. |
|  | Azithromycin | macrolide 🡪 azalide |  | Inhibit protein synthesis, reversible binding to 50S ribosomal subunit, |
|  | - sulfa | sulfonamide |  | Interfere with folic acid synthesis, competition for the enzyme dihydropteorate synthetase |
|  | - quinolone | quinolone |  | Inhibits DNA gyrase |
|  | Colistin | cationic polypeptide | ER response | Solubilize cell membrane through a detergent like mechanism. |
|  | Meropenem | beta-lactam 🡪 carbapenem | ER response | Inhibits cell wall synthesis, penetrates cell wall to reach penicillin-binding-protein targets. |
| M-1 | Aztreonam | monobactam |  | Inhibit synthesis of bacteria cell wall, binds to and inactivates penicillin-binding-protein-3. |
|  | Azithromycin | macrolide 🡪 azalide |  | Inhibit protein synthesis, reversible binding to 50S ribosomal subunit, |
|  | - quinolone | quinolone |  | Inhibits DNA gyrase |
|  | - sulfa | sulfonamide |  | Interfere with folic acid synthesis, competition for the enzyme dihydropteorate synthetase |
| M-6 | Azithromycin | macrolide 🡪 azalide |  | Inhibit protein synthesis, reversible binding to 50S ribosomal subunit, |
|  | - sulfa | sulfonamide |  | Interfere with folic acid synthesis, competition for the enzyme dihydropteorate synthetase |
|  | - quinolone | quinolone |  | Inhibits DNA gyrase |
|  | Meropenem | beta-lactam | ER response | Inhibits cell wall synthesis, penetrates cell wall to reach penicillin-binding-protein targets. |
| M-24 | Doxycycline | tetracycline |  | Inhibit protein synthesis, reversible binding to 30S ribosomal subunit and possibly 50S. |
|  | Ciprofloxacin | fluoroquinolone |  | Inhibits topoisomerase II (DNA gyrase) and topoisomerase IV |

**Supplemental Table 1E**. Metagenome and metatranscriptome sequencing overview for CF01 sputum samples. All libraries were sequenced on the Illumina platform. The Nextera library prep kit was used for all metagenomes whereas TruSeq was used for metatranscriptomes.

| **Day** | **Status** | **Library type** | **File name** | **SRA ID** |
| --- | --- | --- | --- | --- |
| D-724 | Exacerbation | metagenome | polihed_CF01mgD724.fasta | SAMN10605062 |
| D-723 | Exacerbation | metagenome | polished_CF01mgD723.fasta | SAMN10605061 |
| D-722 | Exacerbation | metagenome | polished_CF01mgD722.fasta | SAMN10605060 |
| D-721 | Exacerbation | metagenome | polished_CF01mgD721.fasta | SAMN10605059 |
| D-720 | Exacerbation | metagenome | polished_CF01mgD720.fasta | SAMN10605058 |
| D-719 | Exacerbation | metagenome | polished_CF01mgD719.fasta | SAMN10605057 |
| D-718 | Exacerbation | metagenome | polished_CF01mgD718.fasta | SAMN10605056 |
| D-409 | Stable | metagenome | polished_CF01mgD409.fasta | SAMN10605055 |
| D-286 | Stable | metagenome | polished_CF01mgD286.fasta | SAMN10605054 |
| D-8 | Exacerbation | metagenome | polished_CF01mgD8.fasta | SAMN10605053 |
| D-303 | Stable | metatranscriptome | polished_CF01mtD303.fasta | SAMN10605052 |
| D-279 | Stable | metatranscriptome | polished_CF01mtD279.fasta | SAMN10605051 |
| D-8 | Exacerbation | metatranscriptome | polished_CF01mtD8.fasta | SAMN10605050 |
| D-7 | Exacerbation | metatranscriptome | polished_CF01mtD7.fasta | SAMN10605049 |
