# Supplementary material for: Cystic Fibrosis Rapid Response: Translating Multi-omics Data into Clinically Relevant Information
Source: mBio. 2019 Apr 16;10(2):e00431-19. doi: 10.1128/mBio.00431-19 (PMC6469968; doi:10.1128/mBio.00431-19)
Supplement: FIG S1 [file mBio.00431-19-sf001.pdf]

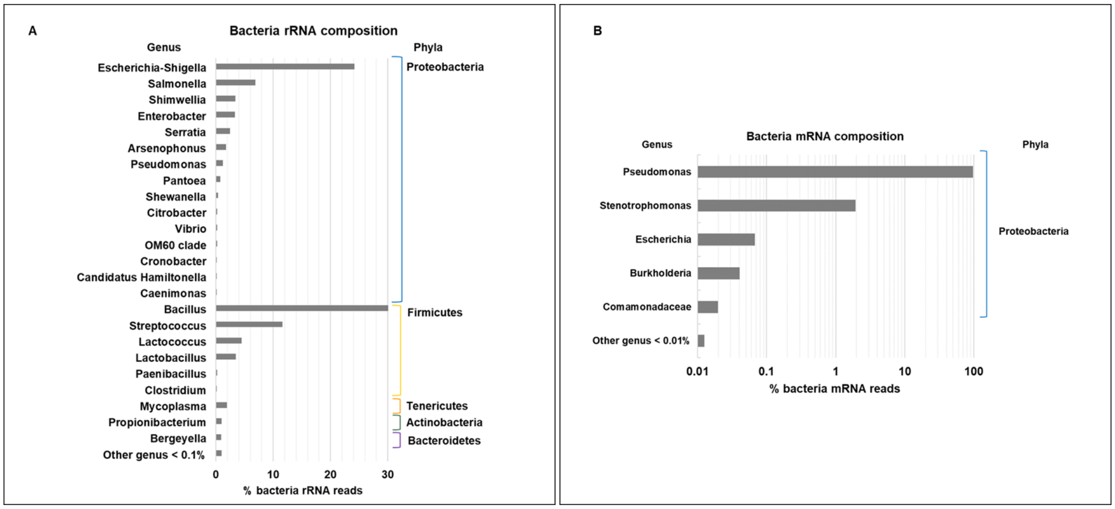

Supplemental Figure 1. Total bacterial RNA composition during a fatal exacerbation (sample D-8). A) Bacterial ribosomal RNA composition was assigned with BLASTn against the SILVA SSU database, with an E-value cutoff of 0.001. The best hit from 10,000 subsample replicates was used. Results are shown at the genus level. B) Bacterial non-ribosomal RNA composition at genus level assigned by BLASTn vs NT with an E-value cutoff of 0.001. The best hit was selected.
