# Supplementary material for: Cystic Fibrosis Rapid Response: Translating Multi-omics Data into Clinically Relevant Information
Source: mBio. 2019 Apr 16;10(2):e00431-19. doi: 10.1128/mBio.00431-19 (PMC6469968; doi:10.1128/mBio.00431-19)
Supplement: FIG S2 [file mBio.00431-19-sf002.pdf]

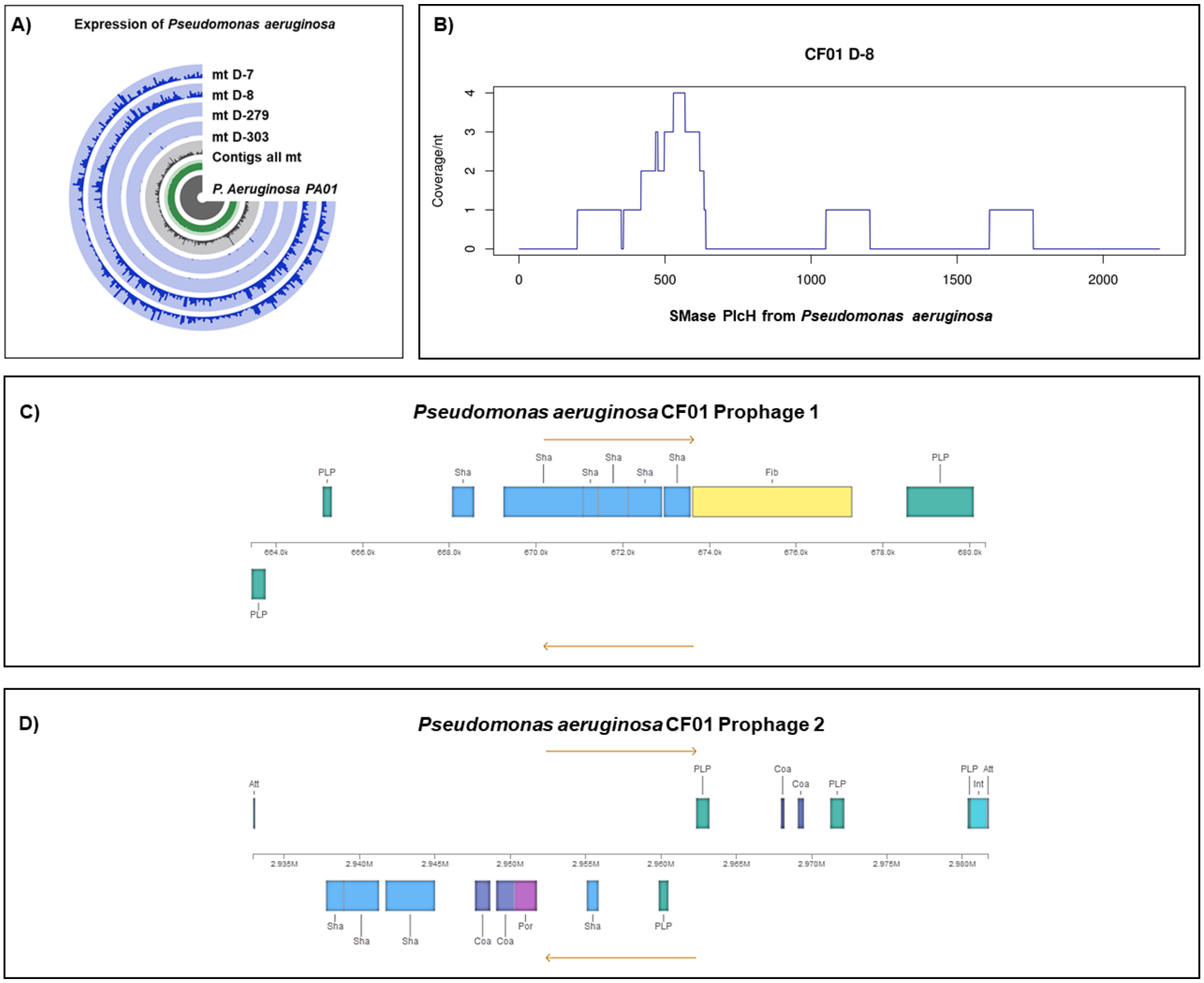

Supplemental Figure 2. A) *P. aeruginosa* gene expression during a stable period (D-279 & D-303) and fatal exacerbation (D-7 & D-8) based on fragment recruitment to the *P. aeruginosa* PAO1 reference genome. All microbial reads from metatranscriptomes D-303, D-279, D-8, and D-7 were individually mapped to the reference genome with SMALT using an identity cutoff 80%. Reads from samples D-8 and D-7 were *denovo* assembled into contigs. Contigs were mapped to the reference genome using SMALT with an identity cutoff 80%. B) *P. aeruginosa* SMase *plcH* coverage plot. Reads from metatranscriptome D-8 were mapped to the gene PlcH using SMALT at 80% identity cutoff. C) Predicted prophages from assembled genome *P. aeruginosa* CF01. Prophages were predicted using the online version of PHASTER. Protein annotations for partial prophage 1. PLP: phage like protein, Sha: tail

protein, Fib: fiber protein. D) Predicted prophages from assembled genome *P. aeruginosa* CF01. Prophages were predicted using the online version of PHASTER Protein annotations for complete prophage 2. PLP: phage like protein, Sha: tail protein, Fib: fiber protein, Coa: coat protein, Por: portal protein, Att: attachment site.
