# Supplementary material for: Cystic Fibrosis Rapid Response: Translating Multi-omics Data into Clinically Relevant Information
Source: mBio. 2019 Apr 16;10(2):e00431-19. doi: 10.1128/mBio.00431-19 (PMC6469968; doi:10.1128/mBio.00431-19)
Supplement: TABLE S2 [file mBio.00431-19-st002.docx]

**Supplemental Table 2A**. Comparison of molecules spectra between non-exacerbation samples (D-426 to D-248) and exacerbation sample D-8. P-values were calculated from a single tail normal distribution (pnorm function in R).

| **Molecule** | **p-value** | **z score** |
| --- | --- | --- |
| Globotriaosylceramide | ~0 | 512960.92 |
| Lactosylceramide | ~0 | 44.60 |
| Sphingomyelin | 0.90 | -1.28 |

**Supplemental Table 2B**. Comparison of number of specific bacteria spectra between non-exacerbation samples (D-426 to D-248) and exacerbation sample D-8. P-values were calculated from a single tail normal distribution (pnorm function in R).

| **Bacteria spectra in D-8** | **CF strain ID** | **p-value** | **z score** |
| --- | --- | --- | --- |
| *Escherichia coli* | VVP427 | 1.99e-17 | 8.41 |
| *Enterococcus sp.* | VVP100 | 0.043 | 1.71 |
| *Pseudomonas aeruginosa* | VVP172 | 3.38e-69 | 17.5 |
| *Staphylococcus aureus* | VVP270 | 2.58e-05 | 4.04 |
| *Stenotrophomonas maltophilia* | VVP327 | 0.005 | 2.51 |
| *Streptococcus sp.* | VVP047 | 2.39e-14 | 7.53 |
