# Supplementary material for: Cystic Fibrosis Rapid Response: Translating Multi-omics Data into Clinically Relevant Information
Source: mBio. 2019 Apr 16;10(2):e00431-19. doi: 10.1128/mBio.00431-19 (PMC6469968; doi:10.1128/mBio.00431-19)
Supplement: TABLE S3 [file mBio.00431-19-st003.docx]

**Supplemental Table 3A**. Antibiotic Resistance Genes present in exacerbation metatranscriptomes. Contigs assembled using D-8 and D-7 metatranscriptomes were screened for antibiotic resistance genes using CARD. Hits with an identity > 80% are shown as well as the prevalence in sequenced genomes and plasmids of 79 important pathogens as reported in CARD.

| **ARO**  **ID** | **ARO name** | **ARO**  **category** | **Prevalence**  **in species** | **Genera** | **cut off** | **e-value** | **identity** | **bit score** |
| --- | --- | --- | --- | --- | --- | --- | --- | --- |
| ARO:3000806 | **MexG** | efflux pump complex or subunit conferring antibiotic resistance | *P. aeruginosa* 99.1% | Pseudomonas | Perfect | 1.1E-102 | 100.00 | 291.20 |
| ARO:3000378 | **MexB** |  | *P. aeruginosa* 4.1% | Pseudomonas | Strict | 0 | 99.81 | 2118.58 |
| ARO:3000377 | **MexA** |  | *P. aeruginosa* 100% | Pseudomonas | Strict | 0 | 99.73 | 753.44 |
| ARO:3000379 | **OprM** |  | *P. aeruginosa* 100% | Pseudomonas | Loose | 8.3E-130 | 100.00 | 374.40 |
| ARO:3000167 | **tet(C)** |  | *Achromobacter xylosoxidans* 14.2% | Achromobacter | Loose | 1.5E-61 | 98.94 | 186.81 |
| ARO:3000809 | **OpmD** |  | *P. aeruginosa* 98.3% | Pseudomonas | Loose | 0.1801 | 81.82 | 24.64 |
| ARO:3000800 | **MexC** |  | *P. aeruginosa* 100% | Pseudomonas | Loose | 1.6291 | 100.00 | 23.87 |
| ARO:3000808 | **MexI** |  | *P. aeruginosa* 99.1% | Pseudomonas | Loose | 0.9241 | 83.33 | 23.10 |
| ARO:3000979 | **TEM-116** | antibiotic inactivation enzyme; determinant of beta-lactam resistance | Broad spectrum |  | Perfect | 0 | 100.00 | 591.27 |
| ARO:3002500 | **PDC-3** |  | Broad spectrum, *P. aeruginosa* 17.3% | Pseudomonas | Strict | 0 | 99.75 | 803.13 |
| ARO:3001796 | **OXA-50** |  | *P. aeruginosa* 100% | Pseudomonas | Loose | 1.2E-24 | 97.73 | 89.35 |
| ARO:3002387 | **BEL-3** |  | *P. aeruginosa* | Pseudomonas | Loose | 1.0982 | 80.00 | 22.71 |
| ARO:3001883 | **CTX-M-21** |  | Enterobacteriaceae family |  | Loose | 7.3404 | 80.00 | 20.40 |
| ARO:3001303 | **ErmO** | antibiotic target modifying enzyme; determinant of lincosamide resistance; determinant of macrolide resistance; determinant of streptogramin resistance | *Streptomyces ambofaciens* | Streptomyces | Loose | 5.2861 | 90.00 | 23.10 |
| ARO:3001305 | **ErmU** |  | *Streptomyces lincolnesis* | Streptomyces | Loose | 0.6121 | 88.89 | 22.71 |
| ARO:3003982 | **LlmA 23S rRNA methyltransferase** | antibiotic target modifying enzyme | *Paenibacillus* sp. | Paenibacillus | Loose | 0.5070 | 81.82 | 23.10 |
| ARO:3002544 | **AAC(3)-Xa** | antibiotic inactivation enzyme; determinant of aminoglycoside resistance | *Streptomyces griseus* | Streptomyces | Loose | 0.1084 | 81.82 | 25.41 |
| ARO:3003069 | **vanXYG** | determinant of resistance to glycopeptide antibiotics; antibiotic resistance gene cluster, cassette, or operon; gene conferring antibiotic resistance via molecular bypass | *Clostridium difficile* 88.8% | Clostridium | Loose | 2.5214 | 85.71 | 22.33 |
| ARO:3003839 | **Mrx** | determinant of macrolide resistance; antibiotic inactivation enzyme | *Morganella morganii* 14.2% | Morganella | Loose | 8.3714 | 80.00 | 19.63 |
| ARO:3003105 | **dfrA3** | antibiotic target replacement protein; determinant of diaminopyrimidine resistance | *E. coli* | Escherichia | Loose | 4.8908 | 87.50 | 20.02 |
| ARO:3000186 | **tetM** | antibiotic target protection protein; determinant of tetracycline resistance | *Clostridium difficile* 11.1% | Clostridium | Loose | 5.0391 | 80.00 | 20.79 |

**Supplemental Table 3B**. Genes that are predicted to encode resistance to antibiotics and that were present in *Pseudomonas* contigs assembled from metatranscriptome reads sampled during the exacerbation. Contigs assembled using D-8 and D-7 metatranscriptomes were annotated using the PATRIC webserver. Subsystems annotations under subclass “resistance to antibiotics and toxic compounds” and “multidrug efflux systems” are shown. Subsystems classified as antibiotics targets are not shown.

| Subclass | Subsystem Name | Role name | PATRIC ID |
| --- | --- | --- | --- |
| Resistance to antibiotics and toxic compounds | Beta-lactamases Ambler class C | Class C beta-lactamase (EC 3.5.2.6) => PDC family | fig\|287.6648.peg.618 |
| Resistance to antibiotics and toxic compounds | Beta-lactamases Ambler class D | Class D beta-lactamase (EC 3.5.2.6) => OXA-50 family, oxacillin-hydrolyzing | fig\|287.6648.peg.2588 |
| Resistance to antibiotics and toxic compounds | Beta-lactamases Ambler class D | Class D beta-lactamase (EC 3.5.2.6) => OXA-50 family, oxacillin-hydrolyzing | fig\|287.6648.peg.2650 |
| Resistance to antibiotics and toxic compounds | Beta-lactamases Ambler class D | Class D beta-lactamase (EC 3.5.2.6) => OXA-50 family, oxacillin-hydrolyzing | fig\|287.6648.peg.3724 |
| Resistance to antibiotics and toxic compounds | Chloramphenicol resistance | Chloramphenicol O-acetyltransferase (EC 2.3.1.28) => CatB family | fig\|287.6648.peg.2030 |
| Resistance to antibiotics and toxic compounds | Copper homeostasis: copper tolerance | Copper homeostasis protein CutE | fig\|287.6648.peg.68 |
| Resistance to antibiotics and toxic compounds | Copper homeostasis: copper tolerance | Magnesium and cobalt efflux protein CorC | fig\|287.6648.peg.1054 |
| Resistance to antibiotics and toxic compounds | Copper homeostasis: copper tolerance | Copper homeostasis protein CutE | fig\|287.6648.peg.2663 |
| Resistance to antibiotics and toxic compounds | Copper homeostasis: copper tolerance | Copper homeostasis protein CutE | fig\|287.6648.peg.2756 |
| Resistance to antibiotics and toxic compounds | Copper homeostasis: copper tolerance | Magnesium and cobalt efflux protein CorC | fig\|287.6648.peg.3966 |
| Resistance to antibiotics and toxic compounds | Fosfomycin resistance | Fosfomycin resistance protein FosA | fig\|287.6648.peg.4961 |
| Resistance to antibiotics and toxic compounds | Fusidic acid resistance | Translation elongation factor G | fig\|287.6648.peg.486 |
| Resistance to antibiotics and toxic compounds | Fusidic acid resistance | Translation elongation factor G | fig\|287.6648.peg.5510 |
| Resistance to antibiotics and toxic compounds | Inner membrane proteins of MarC family, not involved in antibiotic resistance | UPF0056 inner membrane protein MarC | fig\|287.6648.peg.2411 |
| Resistance to antibiotics and toxic compounds | Inner membrane proteins of MarC family, not involved in antibiotic resistance | UPF0056 inner membrane protein MarC | fig\|287.6648.peg.3389 |
| Resistance to antibiotics and toxic compounds | Inner membrane proteins of MarC family, not involved in antibiotic resistance | MarC family integral membrane protein | fig\|287.6648.peg.4551 |
| Resistance to antibiotics and toxic compounds | Listeria Pathogenicity Island LIPI-1 extended | Broad-substrate range phospholipase C (EC 3.1.4.3) | fig\|287.6648.peg.4702 |
| Resistance to antibiotics and toxic compounds | Mupirocin resistance | Isoleucyl-tRNA synthetase (EC 6.1.1.5) | fig\|287.6648.peg.310 |
| Resistance to antibiotics and toxic compounds | Mupirocin resistance | Isoleucyl-tRNA synthetase (EC 6.1.1.5) | fig\|287.6648.peg.443 |
| Resistance to antibiotics and toxic compounds | Mupirocin resistance | Isoleucyl-tRNA synthetase (EC 6.1.1.5) | fig\|287.6648.peg.2630 |
| Resistance to antibiotics and toxic compounds | Polymyxin resistance, lipid A modifications with phosphoethanolamine | Lipid A phosphoethanolamine transferase, putative | fig\|287.6648.peg.365 |
| Resistance to antibiotics and toxic compounds | Polymyxin resistance, lipid A modifications with phosphoethanolamine | Phosphoethanolamine transferase EptC [E.coli], specific for LPS heptose I residue | fig\|287.6648.peg.2825 |
| Resistance to antibiotics and toxic compounds | Polymyxin resistance, lipid A modifications with phosphoethanolamine | Lipid A 4'-phosphatase LpxF-like, putative | fig\|287.6648.peg.3843 |
| Resistance to antibiotics and toxic compounds | Polymyxin resistance, lipid A modifications with phosphoethanolamine | Lipid A phosphoethanolamine transferase, putative | fig\|287.6648.peg.4275 |
| Resistance to antibiotics and toxic compounds | Polymyxin resistance, lipid A modifications with phosphoethanolamine | Phosphoethanolamine transferase EptC [E.coli], specific for LPS heptose I residue | fig\|287.6648.peg.4470 |
| Resistance to antibiotics and toxic compounds | Polymyxin resistance, lipid A modifications with phosphoethanolamine | Lipid A 4'-phosphatase LpxF-like, putative | fig\|287.6648.peg.4493 |
| Resistance to antibiotics and toxic compounds | Polymyxin resistance, lipid A modifications with phosphoethanolamine | Lipid A phosphoethanolamine transferase, putative | fig\|287.6648.peg.4610 |
| Resistance to antibiotics and toxic compounds | Polymyxin resistance, lipid A modifications with phosphoethanolamine | Phosphoethanolamine transferase EptC [E.coli], specific for LPS heptose I residue | fig\|287.6648.peg.5023 |
| Resistance to antibiotics and toxic compounds | Polymyxin resistance, lipid A modifications with phosphoethanolamine | Phosphoethanolamine transferase EptC [E.coli], specific for LPS heptose I residue | fig\|287.6648.peg.5058 |
| Resistance to antibiotics and toxic compounds | Resistance to Daptomycin | Glycerophosphoryl diester phosphodiesterase (EC 3.1.4.46) | fig\|287.6648.peg.242 |
| Resistance to antibiotics and toxic compounds | Resistance to Daptomycin | Glycerophosphoryl diester phosphodiesterase (EC 3.1.4.46) | fig\|287.6648.peg.3185 |
| Resistance to antibiotics and toxic compounds | Resistance to Daptomycin | CDP-diacylglycerol--glycerol-3-phosphate 3-phosphatidyltransferase (EC 2.7.8.5) | fig\|287.6648.peg.4060 |
| Resistance to antibiotics and toxic compounds | Resistance to Daptomycin | Cardiolipin synthase, bacterial type ClsA | fig\|287.6648.peg.4524 |
| Resistance to antibiotics and toxic compounds | Resistance to Daptomycin | Glycerophosphoryl diester phosphodiesterase (EC 3.1.4.46) | fig\|287.6648.peg.5171 |
| Resistance to antibiotics and toxic compounds | Resistance to Daptomycin | CDP-diacylglycerol--glycerol-3-phosphate 3-phosphatidyltransferase (EC 2.7.8.5) | fig\|287.6648.peg.5534 |
| Resistance to antibiotics and toxic compounds | Resistance to Triclosan | Enoyl-[acyl-carrier-protein] reductase [NADH] (EC 1.3.1.9), FabV => refractory to triclosan | fig\|287.6648.peg.676 |
| Resistance to antibiotics and toxic compounds | Resistance to Triclosan | Enoyl-[acyl-carrier-protein] reductase [NADH] (EC 1.3.1.9) | fig\|287.6648.peg.4417 |
| Resistance to antibiotics and toxic compounds | Resistance to Triclosan | Enoyl-[acyl-carrier-protein] reductase [NADH] (EC 1.3.1.9) | fig\|287.6648.peg.5819 |
| Multidrug efflux systems | MexXY System of Pseudomonas aeruginosa | Multidrug efflux system, inner membrane proton/drug antiporter (RND type) => MexY of MexXY/AxyXY | fig\|287.6648.peg.785 |
| Multidrug efflux systems | MexXY System of Pseudomonas aeruginosa | Multidrug efflux system, membrane fusion component => MexX of of MexXY/AxyXY | fig\|287.6648.peg.1128 |
| Multidrug efflux systems | MexXY System of Pseudomonas aeruginosa | Multidrug efflux system, inner membrane proton/drug antiporter (RND type) => MexY of MexXY/AxyXY | fig\|287.6648.peg.1426 |
| Multidrug efflux systems | MexXY System of Pseudomonas aeruginosa | Multidrug efflux system, inner membrane proton/drug antiporter (RND type) => MexY of MexXY/AxyXY | fig\|287.6648.peg.5767 |
| Multidrug efflux systems | MexXY System of Pseudomonas aeruginosa | Multidrug efflux system, membrane fusion component => MexX of of MexXY/AxyXY | fig\|287.6648.peg.6097 |
| Multidrug efflux systems | Tripartite multidrug efflux systems (of RND type) in Pseudomonas | Type I secretion outer membrane protein, TolC family | fig\|287.6648.peg.989 |
| Multidrug efflux systems | Tripartite multidrug efflux systems (of RND type) in Pseudomonas | Multidrug efflux system, inner membrane proton/drug antiporter (RND type) => MexD of MexCD-OprJ system | fig\|287.6648.peg.2027 |
| Multidrug efflux systems | Tripartite multidrug efflux systems (of RND type) in Pseudomonas | Multidrug efflux system MdtABC-TolC, inner-membrane proton/drug antiporter MdtB (RND type) | fig\|287.6648.peg.2490 |
| Multidrug efflux systems | Tripartite multidrug efflux systems (of RND type) in Pseudomonas | Type I secretion outer membrane protein, TolC family | fig\|287.6648.peg.2494 |
| Multidrug efflux systems | Tripartite multidrug efflux systems (of RND type) in Pseudomonas | Multidrug efflux system, inner membrane proton/drug antiporter (RND type) => MexD of MexCD-OprJ system | fig\|287.6648.peg.2648 |
| Multidrug efflux systems | Tripartite multidrug efflux systems (of RND type) in Pseudomonas | Multidrug efflux system MdtABC-TolC, inner-membrane proton/drug antiporter MdtB (RND type) | fig\|287.6648.peg.3159 |
| Multidrug efflux systems | Tripartite multidrug efflux systems (of RND type) in Pseudomonas | Multidrug efflux system MdtABC-TolC, membrane fusion component MdtA | fig\|287.6648.peg.3395 |
| Multidrug efflux systems | Tripartite multidrug efflux systems (of RND type) in Pseudomonas | Multidrug efflux system, outer membrane factor lipoprotein => OprJ of MexCD-OprJ system | fig\|287.6648.peg.3462 |
| Multidrug efflux systems | Tripartite multidrug efflux systems (of RND type) in Pseudomonas | Multidrug efflux system, inner membrane proton/drug antiporter (RND type) => MexD of MexCD-OprJ system | fig\|287.6648.peg.3649 |
| Multidrug efflux systems | Tripartite multidrug efflux systems (of RND type) in Pseudomonas | Multidrug efflux system, membrane fusion component => MexC of MexCD-OprJ system | fig\|287.6648.peg.3928 |
| Multidrug efflux systems | Tripartite multidrug efflux systems (of RND type) in Pseudomonas | Type I secretion outer membrane protein, TolC family | fig\|287.6648.peg.4043 |
| Multidrug efflux systems | Tripartite multidrug efflux systems (of RND type) in Pseudomonas | Multidrug efflux system MdtABC-TolC, membrane fusion component MdtA | fig\|287.6648.peg.4343 |
| Multidrug efflux systems | Tripartite multidrug efflux systems (of RND type) in Pseudomonas | Multidrug efflux system MdtABC-TolC, inner-membrane proton/drug antiporter MdtC (RND type) | fig\|287.6648.peg.4598 |
| Multidrug efflux systems | Tripartite multidrug efflux systems (of RND type) in Pseudomonas | Multidrug efflux system, inner membrane proton/drug antiporter (RND type) => MexD of MexCD-OprJ system | fig\|287.6648.peg.4797 |
| Multidrug efflux systems | Tripartite multidrug efflux systems (of RND type) in Pseudomonas | Outer membrane channel TolC (OpmH) | fig\|287.6648.peg.4882 |
| Multidrug efflux systems | Tripartite multidrug efflux systems (of RND type) in Pseudomonas | Multidrug efflux system MdtABC-TolC, inner-membrane proton/drug antiporter MdtB (RND type) | fig\|287.6648.peg.5150 |
| Multidrug efflux systems | Tripartite multidrug efflux systems (of RND type) in Pseudomonas | Multidrug efflux system MdtABC-TolC, membrane fusion component MdtA | fig\|287.6648.peg.5156 |
| Multidrug efflux systems | Tripartite multidrug efflux systems (of RND type) in Pseudomonas | Outer membrane channel TolC (OpmH) | fig\|287.6648.peg.5219 |
| Multidrug efflux systems | Tripartite multidrug efflux systems (of RND type) in Pseudomonas | Multidrug efflux system, inner membrane proton/drug antiporter (RND type) => MexD of MexCD-OprJ system | fig\|287.6648.peg.5229 |
| Multidrug efflux systems | Tripartite multidrug efflux systems (of RND type) in Pseudomonas | Multidrug efflux system, inner membrane proton/drug antiporter (RND type) => MexD of MexCD-OprJ system | fig\|287.6648.peg.5428 |
| Multidrug efflux systems | Tripartite multidrug efflux systems (of RND type) in Pseudomonas | Type I secretion outer membrane protein, TolC family | fig\|287.6648.peg.5603 |
| Multidrug efflux systems | Tripartite multidrug efflux systems (of RND type) MdtABC-TolC | Multidrug efflux system MdtABC-TolC, inner-membrane proton/drug antiporter MdtB (RND type) | fig\|287.6648.peg.2490 |
| Multidrug efflux systems | Tripartite multidrug efflux systems (of RND type) MdtABC-TolC | Multidrug efflux system MdtABC-TolC, inner-membrane proton/drug antiporter MdtB (RND type) | fig\|287.6648.peg.3159 |
| Multidrug efflux systems | Tripartite multidrug efflux systems (of RND type) MdtABC-TolC | Multidrug efflux system MdtABC-TolC, membrane fusion component MdtA | fig\|287.6648.peg.3395 |
| Multidrug efflux systems | Tripartite multidrug efflux systems (of RND type) MdtABC-TolC | Multidrug efflux system MdtABC-TolC, membrane fusion component MdtA | fig\|287.6648.peg.4343 |
| Multidrug efflux systems | Tripartite multidrug efflux systems (of RND type) MdtABC-TolC | Multidrug efflux system MdtABC-TolC, inner-membrane proton/drug antiporter MdtC (RND type) | fig\|287.6648.peg.4598 |
| Multidrug efflux systems | Tripartite multidrug efflux systems (of RND type) MdtABC-TolC | Outer membrane channel TolC (OpmH) | fig\|287.6648.peg.4882 |
| Multidrug efflux systems | Tripartite multidrug efflux systems (of RND type) MdtABC-TolC | Outer membrane factor (OMF) lipoprotein associated wth MdtABC efflux system | fig\|287.6648.peg.5048 |
| Multidrug efflux systems | Tripartite multidrug efflux systems (of RND type) MdtABC-TolC | Multidrug efflux system MdtABC-TolC, inner-membrane proton/drug antiporter MdtB (RND type) | fig\|287.6648.peg.5150 |
| Multidrug efflux systems | Tripartite multidrug efflux systems (of RND type) MdtABC-TolC | Multidrug efflux system MdtABC-TolC, membrane fusion component MdtA | fig\|287.6648.peg.5156 |
| Multidrug efflux systems | Tripartite multidrug efflux systems (of RND type) MdtABC-TolC | Outer membrane channel TolC (OpmH) | fig\|287.6648.peg.5219 |
| Multidrug efflux systems | Multidrug Resistance, Tripartite Systems Found in Gram Negative Bacteria TEMP | Membrane fusion component of MSF-type tripartite multidrug efflux system | fig\|287.6648.peg.4311 |
