# Supplementary material for: Cystic Fibrosis Rapid Response: Translating Multi-omics Data into Clinically Relevant Information
Source: mBio. 2019 Apr 16;10(2):e00431-19. doi: 10.1128/mBio.00431-19 (PMC6469968; doi:10.1128/mBio.00431-19)
Supplement: FIG S3 [file mBio.00431-19-sf003.pdf]

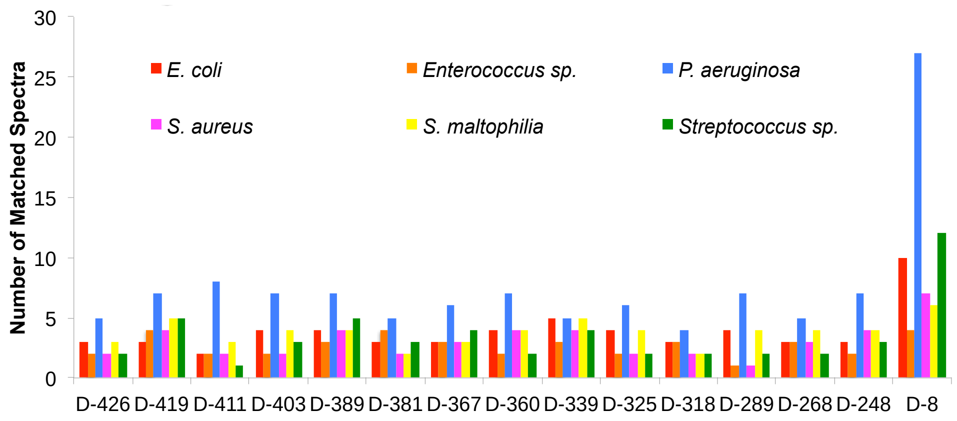

Supplemental Figure 3. Metabolomes from sample D-8 and their comparison to historical samples for CF01. Spectra from CF01 historical samples and fatal exacerbation sample D-8 were mapped to the individual spectra from known bacteria.
