# Supplementary material for: Cystic Fibrosis Rapid Response: Translating Multi-omics Data into Clinically Relevant Information
Source: mBio. 2019 Apr 16;10(2):e00431-19. doi: 10.1128/mBio.00431-19 (PMC6469968; doi:10.1128/mBio.00431-19)
Supplement: FIG S4 [file mBio.00431-19-sf004.pdf]

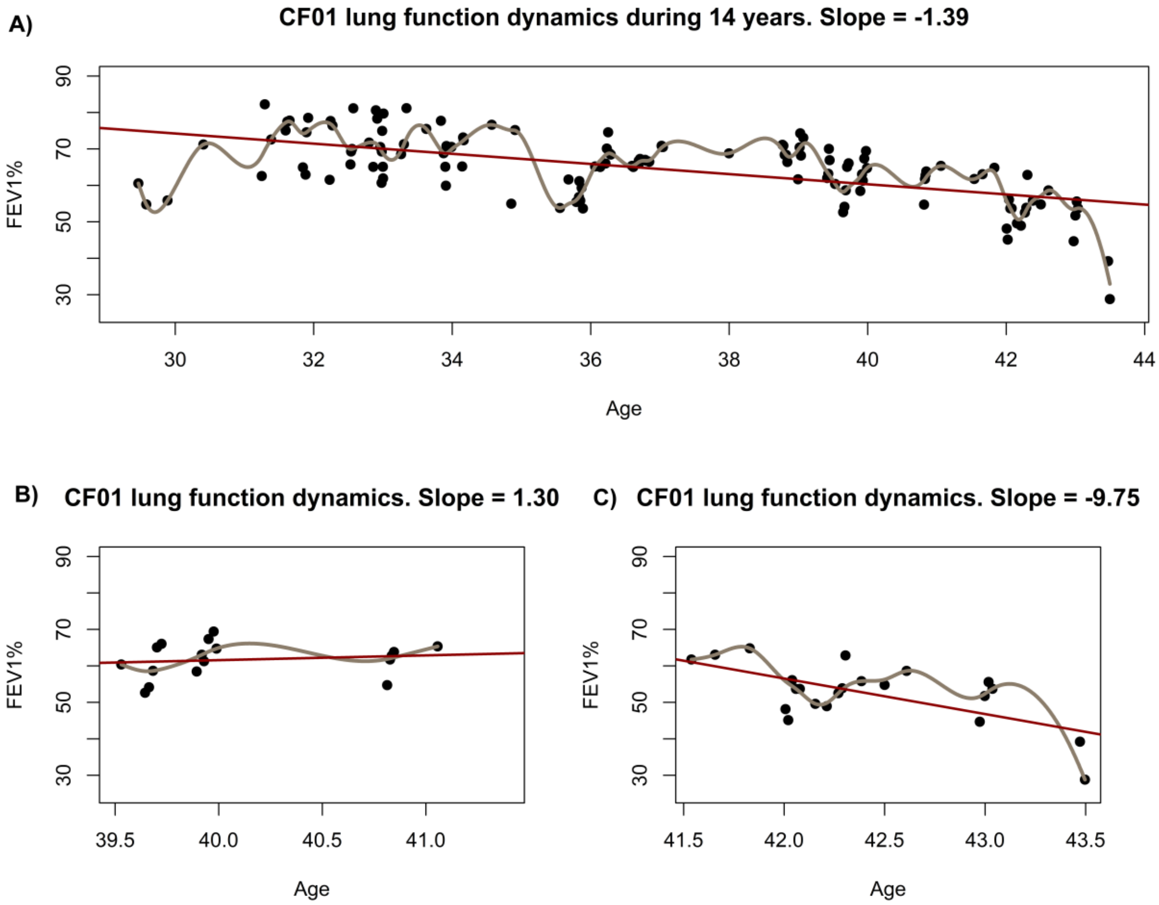

Supplemental Figure 4. A) Percentage of predicted FEV1 of patient CF01 for 14 years. B) Percentage of predicted FEV1 of patient CF01 for years 4 and 3 before death. C) Percentage of predicted FEV1 of patient CF01 for last two years of life. In all panels measurements obtained are represented by black dots. The grey line is the calculated spline as described by Conrad et al. (51). The red line is the fitting of a linear model for the measurements shown in each panel. The slope of the linear model fitting in shown in the header of each panel as the slope.
