# Supplementary material for: Cystic Fibrosis Rapid Response: Translating Multi-omics Data into Clinically Relevant Information
Source: mBio. 2019 Apr 16;10(2):e00431-19. doi: 10.1128/mBio.00431-19 (PMC6469968; doi:10.1128/mBio.00431-19)
Supplement: FIG S5 [file mBio.00431-19-sf005.pdf]

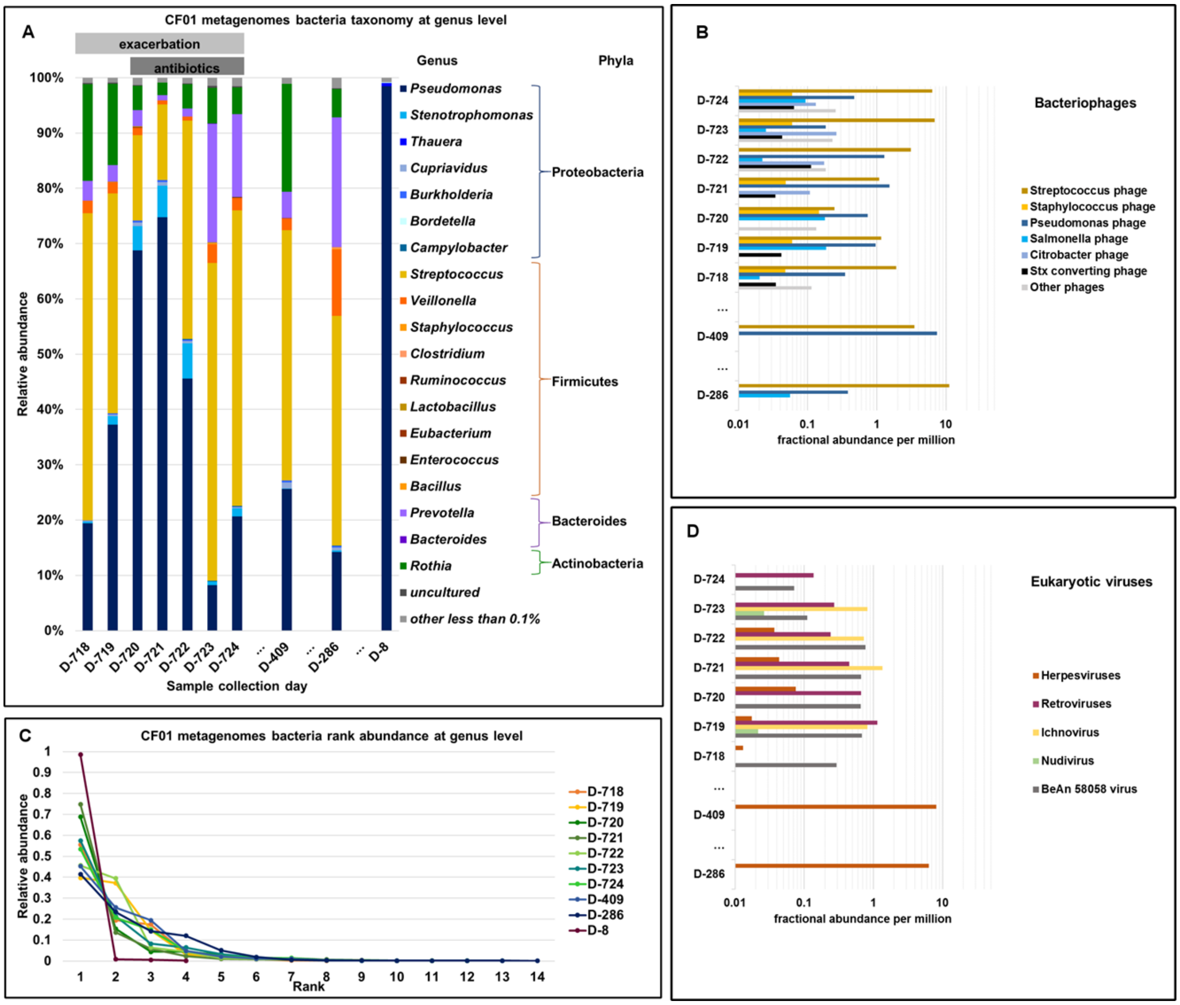

Supplemental Figure 5. Metagenomic analysis was performed from sputum samples collected over a seven-day exacerbation period, during a subsequent stable period of 10 to 14 months and fatal exacerbation. A) Bacterial taxonomy was obtained at the genus level using BLASTn against NT. Relative abundances were calculated for genera whose abundances were greater than or equal to 0.1%. The phylum to which each genus belongs is indicated by a similar color gradient. B) The fractional abundances of phages obtained by mapping to ViralRefseq and FRAP normalization. C) Bacteria rank abundance plots for CF01 metagenomes described in panel A. Relative abundances are shown at genus level, genera with a relative abundance lower than 0.1% were not included in the plots D) The

fractional abundances of eukaryotic viruses obtained by mapping to ViralRefseq and FRAP normalization.
