# Supplementary material for: Cystic Fibrosis Rapid Response: Translating Multi-omics Data into Clinically Relevant Information
Source: mBio. 2019 Apr 16;10(2):e00431-19. doi: 10.1128/mBio.00431-19 (PMC6469968; doi:10.1128/mBio.00431-19)
Supplement: FIG S6 [file mBio.00431-19-sf006.pdf]

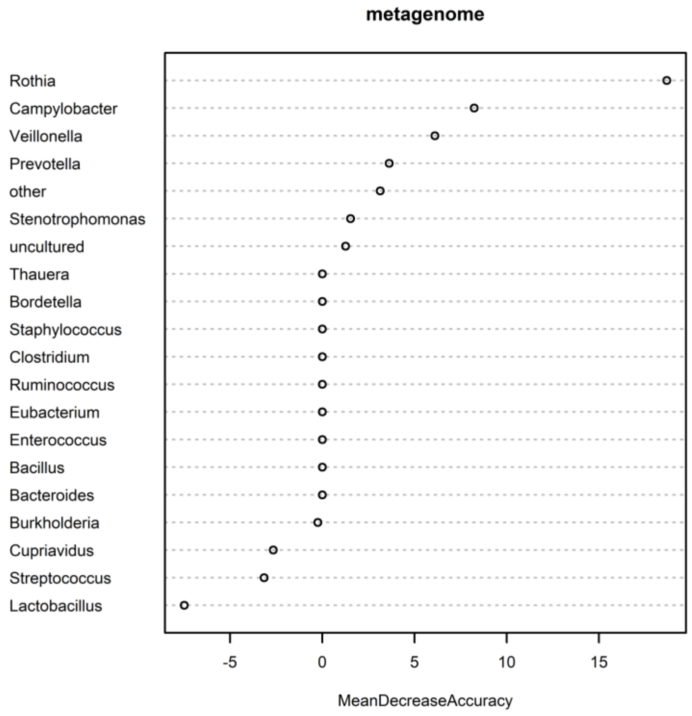

Supplemental Figure 6. Variable importance plot using mean decrease accuracy for a supervised random forest with 5000 trees. Taxonomical relative abundance at genus level in metagenome samples of an exacerbation event two years before CF01’s death. During the exacerbation event, two groups of samples were analyzed: antibiotic treatment and no antibiotic treatment.
