# Supplementary material for: Cystic Fibrosis Rapid Response: Translating Multi-omics Data into Clinically Relevant Information
Source: mBio. 2019 Apr 16;10(2):e00431-19. doi: 10.1128/mBio.00431-19 (PMC6469968; doi:10.1128/mBio.00431-19)
Supplement: FIG S7 [file mBio.00431-19-sf007.pdf]

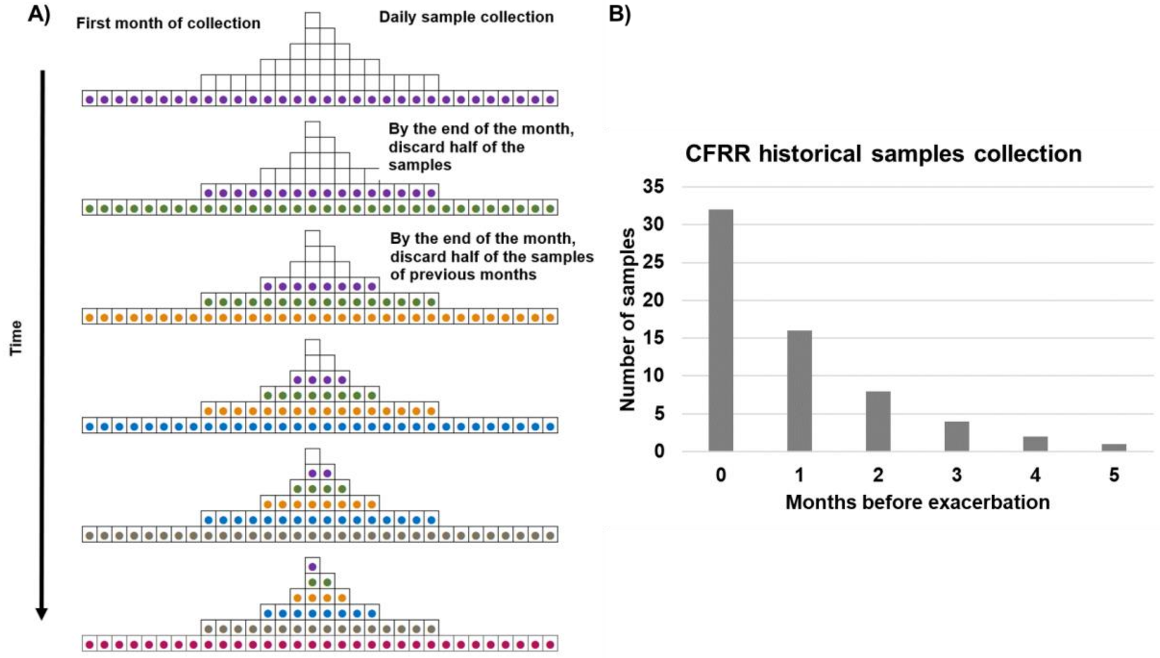

Supplemental Figure 7. Sampling scheme for collection of “historical” sputum samples. A) Proposed at-home sample collection scheme where sputum samples are obtained daily. By the end of the first collection month, half of the samples are discarded (purple dots) and daily collection continues for the new month. By the end of the second month, half of all the previous months samples are discarded (purple and green dots) and so on. B) In an acute exacerbation event, the patient will bring the samples to the clinic and the CFRR methods will be applied to acute exacerbation and historical samples. With the proposed at home collection scheme, a higher sampling density will be obtained in the exacerbation month and less in the months before the exacerbation.
